# "The Hospital" Nursing Mirror

**Published:** 1898-10-15

**Authors:** 


### "The Hospital" Nursing Mirror.

Being the Nursing Section of "The Hospital."

[Contributions for this Section of "The Hospital" should be addressed to the Editor, The Hospital, 28 & 29, Southampton Street, Strand, London, W.C., and should have the word "Nursing" plainly written in left-hand top corner of the envelope.]

### News from the Nursing World.

### MISS ORME'S FAREWELL.

Few are granted the privilege of reviewing twenty-five years of continuous, prosperous work, fewer still have the happiness of rejoicing in the affectionate recognition of their labours by those for whom and with whom they have worked, as well as by those for whose benefit the work was done. Miss Orme closes an active career as matron of the London Temperance Hospital amidst the sincere regret of a multitude of friends. The board of management and medical and surgical staffs of the hospital have borne witness to the excellence of her management, and to the able assiduity with which she discharged her duties; her nurses have testified their affectionate recognition of her kindly thought for their happiness and comfort; and the domestic staff have expressed hearty sorrow at her departure. It is no easy task to take up the work of a popular matron; but Miss Lucas is especially fortunate in having already won the esteem, not only of her predecessor, but of the authorities under whom she serves, and of the staffs over whom she will hold sway. Miss Orme is now taking a prolonged holiday, after which she will devote her energies to the cause of temperance, and especially to the cause of the London Temperance Hospital.

### LADY LEICESTER'S SCHEME.

The Countess of Leicester convened an influential meeting of Norfolk ladies in the Shirehall, Norfolk, on October 1st, for the purpose of considering the best mode of establishing an association to provide cottage nurses for the county. We are sorry that the nurse is not to be, as it was said, a "full blown expert from the London hospitals," but a cottage help with an elementary knowledge of attendance on the sick. Whilst fully acknowledging that a little knowledge may be a useful, as well as a dangerous thing, and that it is infinitely better than no knowledge at all, yet it has still to be proved that the cottage nurse is as fitted for her domestic duties or is even cheaper than her fully trained compeer. In the meantime, whilst it is certain that the best skill is most needed in the village and outlying homes, where the visits of the medical attendant are of necessity few and far between, and the treatment of the case is greatly helped by the accurate reports of an intelligent and well-trained nurse, cottage nurses are certainly better than no nurses at all. They are nevertheless but a poor substitute for those who are fully competent, and they cost nearly as much.

### PLAGUE NURSES IN INDIA.

So many rumours, of a more or less unpleasant nature, are afloat concerning the conditions and regulations under which the nurses sent out by the India Office for nursing the plague in India are engaged, that it seemed worth while to inquire what was known of the state of affairs at the Office. In the first place, the India Office has nothing to do with the rules or arrangements under which the nurses work; the Bombay Government settles all that. No official intimation, therefore, of the new rules, at which so great an outcry has been raised, has been sent home; and although the home authorities are acquainted with them by means of the press, little importance is attached to them, it being thought that very probably local requirements make them desirable, if not necessary. The new regulations have not checked the applications of candidates to have their names put on the reserve list, and many excellently qualified nurses are now awaiting their opportunity for work in the East. No news whatever has been received of the reported strike of the nurses at Rangoon.

### NURSING IN CUMBERLAND.

On Saturday last the first annual meeting of the Cumberland Nursing Association was held in the County Hall, Carlisle, under the presidency of the Countess of Lonsdale. A large and influential gathering assembled to hear the report, which was satisfactory. The Queen's Jubilee Institute has contributed £50 towards the superintendent's salary, and five nurses are engaged under her in the surrounding districts. The superintendent accomplishes her travelling by means of a bicycle, and Lady Lonsdale, who was re-elected president, made known that the gift of second-hand cycles for the use of the nurses would be acceptable.

### VICTORIAN ORDER OF NURSES.

The chief Lady Superintendent of the Victorian Order of Nurses, Ottawa, Canada, is Miss MacLeod. The demand for the nurses in Toronto averages sixty a week, and most of the patients pay a small fee for their services, as they are quite unable to meet the full cost. The nurses receive a special course in district nursing, and several are now in training. Applications have been received from four or five districts for nurses, which will be complied with as soon as the nurses are ready for work. Each district, of course, guarantees the salary of its own nurse.

### MORE KNOWLEDGE.

Whilst on the one hand many advance the claims of the untrained attendant for the sick poor, others urge that the district nurse should not only be a nurse but a sanitarian. Such was the burden of the papers and discussions at the Birmingham Sanitary Institute on the subject of district nursing. Miss M. M. Jenkyn-Brown said that a nurse did not fulfil her mission if she did not convince the friends and relatives of her patients as to the value of cleanliness and fresh air; and Miss Wakeford was in favour of training girls in the knowledge of domestic sanitation and personal hygiene in the elementary schools. Dr. Mary Sturge pleaded for the right of children to be born with sound constitutions, and advocated the instruction of parents in the laws of heredity; she also pointed out the advantage of providing children with playgrounds other than the streets, where the constant warnings of the police occasioned them severe nervous strain. Miss C. J. Wood read a paper on "Village Nursing of Infectious Diseases," and Miss M. E. Tait gave some of her experiences as an officer of the Chesterfield Infant Life Protection Society.

### ANOTHER NURSES' HOME.

Not long since a new home for district nurses was opened in Regent Street under the auspices and control of the Ladies' Sick Nursing Society. The home is comfortably fitted up, and is intended to commemorate the Queen's Jubilee.

### THE REASONS WHY.

Some strictures having been passed on the difficulty of the examination papers set the nurses by Dr. Turner, of Cape Town, that gentleman took the opportunity afforded by the meeting of the Cape Medical Council of explaining why he was not suited to the office of examiner of nurses. In the first place, he considered that an examiner should be in active practice. As it was some time since he had retired, and he was chiefly occupied with the rinderpest, he was not fitted to examine in midwifery, although he was fully competent to do so on sanitary science. The next reason why he was not fitted for this work was that he wanted to go home. In spite of Dr. Turner's protest, however, he does not seem to have lacked ability in the task he repudiates, for of the murmuring candidates between 80 and 90 per cent. passed, and he offered a pertinent remark when he pointed out that the papers should not be signed by the candidate's name, but only by a motto or number only. The result of stringent examinations will be to improve the status of nurses trained in the hospitals of Cape Town.

### INDIAN NURSES.

The following sisters engaged in the Indian Nursing Service have entered for another term of five years: E. M. Carre, J. M. A. Lloyd, and H. A. M. Rait. Each of them will have a year's leave before resuming her duties.

### BROKEN AGREEMENTS.

It is the custom at some hospitals to require from probationers who break their engagement without due cause a forfeit of £5 or £10, and some such rule seems very necessary in the interests of institutions. It is curious to note the elasticity of some people's consciences on such matters. There are, it is to be feared, many women who do not hesitate, should any more profitable opening present itself, to cancel an undertaking made previously, when undeterred by the fear of suffering some pecuniary loss in consequence; and serious inconvenience is not infrequently caused by the failure of accepted candidates to enter upon their trial month at the very last moment, under the apparent impression that because their agreement with the hospital is not signed and sealed they are at liberty to throw up their appointment if so they choose. It cannot be too clearly understood that to do this, except under most extraordinary circumstances, is simply dishonourable. An undertaking to enter a hospital for training in serious work cannot be put aside as easily as an engagement for tea. The trouble caused by having suddenly to find a substitute for a defaulting candidate makes no small addition to the day's work of a busy matron, and it is one which ought not to occur.

### A NEW NURSES' HOME.

The Homœopathic Hospital, Birmingham, is undergoing a series of improvements and alterations. A block for the accommodation of the nurses was opened the other day with some ceremony by Mr. Impey, the chairman, the new home affording the hospital an opportunity of adding to their nursing staff. The bedrooms and sitting-room are comfortably furnished. The future extensions will allow two wards to be added to the hospital, bringing the total number of beds up to fifty.

### CHESTERFIELD NURSES.

The first report of the Chesterfield Victoria Home for Nurses has just been issued. Patients are divided into three classes—those who pay full fees, those who pay reduced fees, and those who pay none. The result is, as might have been expected, the claims of the poor swamp those of the well-to-do; the committee are, therefore, wisely considering ways and means of providing a district nurse for the sole benefit of the destitute. Arrangements will be made for her to reside at the home, but the want of money is keenly felt, and subscribers are invited to put down their names early, in order that the work of the coming winter may be provided for.

### SHORT ITEMS.

On October 28th a lecture on "The Massage of Fractures" will be given at the Trained Nurses' Club, 12, Buckingham Street, by D'Arcy Power, Esq., F.R.C.S., at a quarter to eight p.m. Lectures on Midwifery, in preparation for the L.O.S., are given at the club twice a week, and once a week a lecture on Nursing for Midwives.—A matinée performance, under distinguished patronage, will be given by kind permission of the managers, at the Empire Theatre, Leicester Square, on Thursday, November 10th, in aid of the Queen Victoria Jubilee Institute for Nurses.—The board of management of the Larne Workhouse has convened an early meeting of the Guardians to consider the request of the Local Government Board of Ireland that they should appoint paid attendants in lieu of the pauper nurses at present working there.—Mr. Willet, of Slough, has offered to give a lecture, entitled "Eighteen Hundred Miles up the Yangtse Valley," in aid of the Slough, Upton, and Chalvey Parish Nursing Fund. The offer has been accepted.—The Slough Diamond Jubilee Committee has decided to buy Osborn Villa, to repaint it, and then to place it at the disposal of the Nursing Committee on terms to be arranged hereafter.—The Countess Percy will lend her house, in Grosvenor Square, on October 26th and 27th, for a floral bazaar and concert in aid of the Plaistow Maternity Charity and Nurses' Home.—The Huddersfield Nursing Institution has received a legacy of £10 from the estate of Mr. William Scott.—There is a credit balance of £12 in the annual accounts of the Beauly Nursing Association. The annual meeting has been held recently, over which Lady Lovat presided.—A meeting of the Barry Nursing Association was held lately to consider the offer of the Urban District Council to contribute £70 a year to maintain a nurse who should give her time to the care of fever cases. The offer was refused, as the association has not room to accommodate the nurse, and the sum offered for her maintenance is not large enough. The treasurer of the Association said that it would require £350 to liquidate the debt against the society at the end of the year.—The Helston District Nursing Association has made public its first report. The nurse has given satisfaction, and attends both free cases and those which pay a sum in accordance with their means.

### Antiseptics for Nurses.

By a Medical Woman.

### XX.—PEAT AND OTHER ANTISEPTIC SUBSTANCES.

The disinfectant and deodorant powers of various soils, and especially of peat, have been known and utilised in various ways for many years now. An attempt was also made at the same time to use it for dressing wounds, but it was not as successful in this capacity as had been hoped. The peat when quite dry was so friable that it was reduced to fine powder with very little manipulation, and as there was no means of making it cohere, the only plan was to put it into gauze-bags and use it in the shape of pads. Even in this form, however, its use was attended by much inconvenience, as the fine powder escaped through the gauze, and when the pads were applied it was difficult to get an even surface and a uniform distribution of the peat. Peat is very absorbent, and proved useful as a dressing when there was much discharge; but, according to experiments made by Gaffky, it has only feeble bactericidal powers, and does not completely prevent the development of micro-organisms, though it will retard considerably their multiplication; hence it is an antiseptic, but cannot be regarded as a true disinfectant.

Professor Songka found that the few micro-organisms which peat allows to multiply, belong chiefly to the harmless and indifferent type, and are non-pathogenic. He also found that the soluble substances which can be extracted from peat by soaking it in water have the power of retarding the growth of pathogenic micro-organisms. The drawbacks connected with the use of peat for surgical dressings caused it to be neglected for some years, but recently it has been revived once more, and by special treatment and preparation has been rendered available as an application for wounds. It is now prepared in the form of peat wool or wadding, which is described as quite free from dust, and consisting of the fibres of the moss of which peat is largely composed, which form a brownish mass of innumerable interlaced fibres, and produce a very soft dressing, having great absorbent power, and one which fully saturated has not the same tendency to form hard lumps as wool has under similar circumstances. The wool is very thoroughly sterilised before it is packed, and before use as a surgical dressing it can be steeped in an antiseptic solution. Peat wool is extremely elastic, and is therefore very valuable where an elastic compressing force is required. The other preparation is peat gauze, which is described as being as fine, if not finer, than ordinary gauze, and containing 50 per cent. of peat. It can be used either dry, or wrung out of any antiseptic lotion, as preferred.

Hermitine is the name of a disinfectant solution which is obtained from sea-water by a process known as electrolysis: or for medical purposes it may be produced from a saline solution containing sodium and magnesium chlorides. It is said to destroy instantly all bad smells, and to arrest putrid fermentation. According to Dr. Pioger, of Paris, hermitine is a perfect antiseptic. It owes its disinfectant properties entirely to the liberation of chlorine, which occurs in large quantities. It is a clear, colourless liquid, with a slightly alkaline reaction. Hermitine should only be diluted with water as it is required for use, as it is very unstable in diluted solutions; its strength can be easily regulated by the amount of water added, as it is prepared in solutions of known strength. Recently it has been found that if a small quantity of soda or milk of lime be added to hermitine it does not lose its strength so readily, and a patent has been taken out for this discovery.

Pyroligneous acid is the name given to the products obtained by heating wood in hollow iron cylinders, having a suitable arrangement of coolers or condensers and receivers. The substance thus obtained is of a dark brown colour, with a strong, burnt, acid smell, and a very sour taste. It contains a large quantity of tar and oily matter, from which it is purified to some extent by repeated distillation, but it always remains very impure. It owes its slight antiseptic properties to the presence of tar.

Aminol is a liquid disinfectant and antiseptic. It is a clear (alkaline) fluid with a strong fishy odour, and is, in fact, a solution of ammonia and amines. Aminol possesses only very feeble antiseptic properties, and cannot be regarded as a real disinfectant.

Selana is a natural antiseptic water, which has recently been discovered in Algeria by accident when boring for petroleum. It has been favourably reported on by the Paris Academy of Medicine, and its antiseptic and astringent properties are said to be very remarkable; and to these must be added considerable soothing effects, so that it will probably prove valuable for baths. It contains silica, lime, magnesia, oxide of iron and alumina, sodium chloride, and sulphurous acid.

Tyree's antiseptic powder is said to contain a variety of mineral and vegetable antiseptics. It is a pale pink homogeneous powder, which dissolves readily in water, and is said to be non-irritating and non-toxic, and to possess fairly good antiseptic properties.

Carbo-phenol is a disinfectant powder in which the active ingredient is carbolic acid, and it is said to contain 20 per cent. of the crude carbolic acid from which the phenol has not been removed, and hence is superior to many of the commercial carbolic acid powders, which frequently consist of lime or chalk with the minimum of carbolic acid, and are practically useless as disinfectants. Carbo-phenol does not sink at once in water, having in fact about the same specific gravity, and hence acts more effectually as a disinfectant, and has a distinct advantage over those powders which have a lime or siliceous base. Carbo-phenol cannot be ranked as a very powerful disinfectant, but is very useful for keeping sinks and drains sweet.

Terpinol is described as an actively antiseptic, disinfectant, and deodorant fluid, which is also said to be non-poisonous, and does not stain or destroy articles of clothing. For disinfecting clothing and utensils a solution of 1 part of terpinol in 10 of water is recommended, and after steeping the clothes in this they must be boiled. As the disinfectant properties of terpinol are derived from various species of pine, including Pinus sylvestris, it is certainly non-poisonous and antiseptic, but cannot be recommended as a reliable disinfectant in cases of infectious disease.

Kresyline is one of the most recent modifications of carbolic acid of which there are now so many in the market, and, like these, it owes its antiseptic properties to its coal-tar constituents. It is said to contain all the antiseptic and disinfectant properties of carbolic and cresylic acid, without their disagreeable smell, and to be non-poisonous, non-corrosive, and not to stain. For washing wounds, 1 part kresyline is to be mixed with 100 parts water, whilst for disinfecting clothes and utensils in a sick-room a solution of 20 to 100 parts of water is recommended. Kresyline can be relied on as a fairly good disinfectant.

A patent disinfecting fluid called "Worthall" is a compound apparently of chlorine, and has a strong pungent odour. It is described as an "instantaneous sterilising, disinfecting, and deodorising solution," and does not corrode or stain. The results of experiments on micro-organisms prove " Worthall " to be a reliable and efficient disinfectant, and one that can be recommended for use in cases of infectious disease, being equal in efficiency to carbolic acid. Morever, it is not described as "non-poisonous," which is decidedly in its favour.

Ialine is a new disinfectant prepared from coal-tar and "other ingredients," and claims to contain the really disinfectant principles of tar, such as phenols, carbolic, cresylic, and other acids, without free carbolic acid, and consequently not to possess corrosive and irritant properties. The results of experiments with ialine seem to be most satisfactory, and it can be relied on as an effectual disinfectant.

Oxychlorogine, of which the active principle is chlorine, can also be recommended as a good disinfectant.

### The Silver Anniversary of the London Temperance Hospital.

### PRESENTATIONS TO THE OUTGOING MATRON.

SINGULARLY happy is the London Temperance Hospital in its festivals, and that, on Friday last, celebrating the twenty-fifth anniversary of its inauguration, and at the same time bidding Godspeed to the outgoing matron, Miss Orme, was no exception to the rule. The hospital looked spick and span, and was prettily decorated for the occasion. The reception-room was gay with crimson drugget on the floor, green curtains and white ones at the windows, with plants and flowers—palms, white chrysanthemums, and marguerites, whilst the tea tables were adorned with roses. From six p.m. light refreshments were served, and at seven p.m. Mr. Vesey Strong, vice-chairman of the board of management, took the chair in the regrettable absence of Mr. Cash, the chairman, who was ill. The proceedings opened with prayer, and at the conclusion the assembly joined in the Doxology.

Mr. Vesey Strong said that the absence of Mr. Cash, who had been chairman for the last twenty-five years, occasioned the keenest regret to all, especially as the object of the meeting was to express the indebtedness of the hospital to Miss Orme on her retirement, and to speak the wish felt by everyone who knew her that that retirement might be happy and comfortable. The Chairman then read one or two of the numerous letters of regret, and gave a list of names of those unavoidably absent. Amongst them were those of Lady Battersea, Lady Elizabeth Biddulph, Mr. George Cadbury, &c. Conttnuing, he said that the hospital had advanced to the front rank of London hospitals, a result due in a great measure to Miss Orme, who was entitled to the gratitude of every friend of the institution, and he would call upon five or six gentlemen who had also been five-and-twenty years in the service to express it.

Mr. Dawson Burns, the hon. secretary from the inauguration of the hospital, said that in the beginning two years were spent by the Provisional Committee in seeking a suitable house, which was finally found in Gower Street, and several months were occupied in adapting the building and raising the necessary money. On October 3rd, 1873—a Friday—an opening soirée was held in the building, and on the 6th the first patients were received. The number of beds were then only 17, and as many cases are now received in one year in the present hospital as were admitted during the whole seven and a-half years at the Gower Street house. The east wing of the new hospital was opened in May, 1881, by the then Lord Mayor, and, four or five years later, the west wing was opened by the Archbishop of Canterbury. Through all changes Miss Orme has been matron, and had discharged her multitudinous and multifarious duties with conscientiousness and assiduity. Miss Orme had sent in her resignation, and the board of management, knowing the strain of her position, had reluctantly accepted it. Whatever the hospital owed to the doctors it owed still more to the matron. The board met weekly, and Miss Orme's unfailing report of every detail of the work kept it conversant with all that transpired. The hospital owed much in other ways to Miss Orme. She could represent it in every way. She could not only receive approaches from persons interested in the work, but could approach likely persons on behalf of the hospital, thereby gaining for it some of its warmest supporters. Miss Orme has also the gift of speaking in public, and had repeatedly given addresses which had never failed to be satisfactory. She was also a writer, and there was an admirable epitome of the hospital from her pen. The speaker concluded by saying that he would not bid her farewell in a final, but in a provisional sense.

Dr. Ridge, representing the medical department; Mr. John Mann, one of the first members of the board; Sir John Hutton, and Mr. T. P. Whittaker each spoke in turn of Miss Orme's ability and popularity; and then the Chairman presented her with an illuminated address and a cheque for a hundred and ten guineas on behalf of friends connected with the institution.

Miss Richardson in a few appreciative sentences, asked Miss Orme to accept a silver tea-service from the sisters and nurses.

One of the officials (Mr. Handcock) then presented her with a timepiece from the domestic staff.

Miss Orme expressed her thanks very gracefully. She said that it was impossible to look back over twenty-five years without feeling pride in having worked for such an institution, and without humility in the remembrance of many errors and shortcomings. Authors had the privilege of correcting and revising the proofs of their books, but in the book of one's life no error could be erased, no passage expunged, no explanatory comments interpolated. She had tried to do her best, to do God's will, and so perhaps all the rest might be forgiven. She thanked the board of management for having been very, very good to their "small matron," whom they must at times have found very difficult to understand, the medical and surgical staffs, and the sisters and nurses. The lives of the officers in a hospital, she said, were lived at great pressure, and a matron is not able to spare them always as much as she could wish, but she believed they had always given her credit for being their friend. The domestic staff also strove to do their work as loyally as any other officers in the hospital. The secretary she thanked for his unfailing courtesy and temper, and finally expressed her appreciation of her successor and friend, whom, during the four years they had worked together, she had found both just and kind, and who would use her best endeavours to promote the welfare of the hospital. The board, she continued, seemed to think that when a woman's brain lost its freshness and her hand its cunning that her tongue could go on as freshly as ever. After a rest amidst new scenes and other thoughts she might possibly return and take up the work they had asked of her. She, therefore, wished them all *Adieu*, and, if it were His will, *Au revoir*.

A selection of music and songs was given at ntervals during the evening.

### Training in the Provinces.

(*Continued from page* 16.)

### HULL ROYAL INFIRMARY (188 Beds).

### Terms of Training.

Candidates for training at the Hull Royal Infirmary should be between 23 and 33 years of age. If accepted after one month's trial, they are received for a term of three years' training, a certificate being granted at the satisfactory conclusion of the engagement. Lectures are given to the nurses by the house surgeon and by the matron.

A premium of 15 guineas is required from probationers, and no salary is offered for the first year; £14 per annum is given to second year probationers, and this is increased to £17 during the third year. Nurses who, after their training, join the private staff, receive a salary ranging from £25 to £36; charge nurses in the infirmary are paid at first £25, rising to £30. Laundry and indoor uniform (dresses and caps) are provided by the hospital for nurses and probationers.

A "Nurses' Co-Operation" exists in connection with the private nursing staff, and those nurses who have been attached to the infirmary for four years are allowed to live outside and take their own earnings, subject to a percentage to the hospital for expenses.

Ordinary probationers of good education and general suitability are eligible for promotion to staff appointments, three years' training being in every case an essential qualification. Nurses in their second and third years take four months' night duty each year.

### Hours On and Off Duty.

Nurses and probationers are on duty from 7 a.m. to 9 p.m., with 2 to 2½ hours off duty daily, and time for meals. The hours off duty for probationers are from 2 to 4.30 p.m., or on alternate days either from 5 to 7 p.m. or from 7 to 9 p.m.

Charge nurses' hours are from 8 a.m. to 9 p.m., with two and a half hours off duty each day, taken in the afternoon and evening alternately. Each nurse is allowed one day off a month, from 10 a.m. to 9 p.m., and charge nurses have leave of absence once a month from 5 p.m. on Saturday to 8 a.m. on Monday.

Night nurses are on duty in the wards from 9.5 p.m. to 8 a.m., taking their recreation from 9.30 to 12.15 in the morning.

### Meals.

All meals except luncheon are served in the nurses' dining room. Breakfast for the day probationers is at 6.40 a.m., dinner at 1 p.m., tea at 4.30, and supper at 9.5 p.m. Charge nurses breakfast at 7.30, lunch is served at 10 a.m., dinner at 12.30, tea at 4.30, and supper at 9 p.m. Stores for the nurses in each ward are sent to the ward kitchens for the early luncheon.

### KILMARNOCK INFIRMARY (140 Beds).

### Terms of Training.

Candidates for training at Kilmarnock Infirmary should be between 22 and 30 years of age. They enter the infirmary for a trial of one month, and if considered suitable, are received for a course of three years' training, at the end of which a certificate is granted, after examination. Lectures are given by members of the medical and surgical staff upon anatomy, physiology, &c., during the period of training.

No premium is required from probationers, salary beginning the first year at £12, increasing the second year to £16, and the third year to £20. The salaries of staff nurses range from £22 to £25; of sisters, from £30 to £35.

Probationers who have satisfactorily completed their engagement and gained their certificate, may be promoted to appointments as staff nurses or sisters if they show capacity for the work.

Indoor uniform and laundry are provided by the infirmary, nurses being required to find their own outdoor uniform.

Night duty is taken in rotation for periods of two months.

### Hours On and Off Duty.

The working day is estimated at eleven hours, with time for meals. Two hours daily are allowed for recreation; extra hours or a half-day being granted when specially requested and convenient. On "days off" nurses are on duty till 10 a.m. Three weeks' annual leave is given. Night nurses are on duty from 9 p.m. to 9 a.m.

### Meals.

The hours of meals for day nurses are as follows: Breakfast, 7 a.m.; dinner, 12 and 12.30 p.m.; tea, 5 and 5.30 p.m.; supper, 9 p.m. Afternoon tea is served in the ward kitchens. Butter, tea, sugar, &c., are served out twice daily from the stores.

### LEEDS GENERAL INFIRMARY (394 Beds).

### Terms of Training.

Probationary nurses are admitted for training at the Leeds General Infirmary on one month's trial, and if appointed by the board at the end of this period are required to continue in that capacity for twelve months; then, if considered efficient, they remain in the service of the infirmary as nurses for two years longer. The age for candidates is between 23 and 32. A small salary is given during the three years' training; £10 the first year, £14 the second, and £18 the third. Laundry and indoor uniform are provided by the hospital, the uniform consisting of four print dresses, four caps, six aprons, two belts, and three collars. Nurses or probationers leaving the infirmary are required to give up their uniform. Any nurse breaking her three years' engagement is required to pay a forfeiture of £6, and give three months' notice.

Nurses who remain in the service of the infirmary after the completion of the three years' training are paid a salary of £22. Sisters' salaries range from £30 to £40, according to importance of office and length of service, at the discretion of the board on the advice of the lady superintendent.

Lectures are given to the nurses during their training, and examinations held after each course, the certificate being granted after the completion of the three years' engagement. The fifteen sisters' appointments are filled up as vacancies occur from amongst the best of the probationary nurses who have completed their training.

Nine months' night duty is taken by nurses in their second year.

### Hours On and Off Duty.

Day nurses and probationers' hours on duty in the wards are between 7 a.m. and 9 p.m. During these hours half an hour each is allowed for lunch, dinner, and tea, with two hours off duty daily from 2 to 4 or 5 to 7 on alternate days.

Night nurses are on duty from 8.55 p.m. to 7 a.m., and they take their recreation after their breakfast at 7.10 till 11.30 a.m. A whole day is allowed off duty once a month, night nurses having two nights off every two months. On alternate Sundays sisters, nurses, and probationers alike are off duty from 9 a.m. to 1 p.m., or from 1 p.m. to 5 p.m., and at 5 p.m. for the rest of the day.

Probationers are entitled to 14 days' leave of absence at the end of their first year, and afterwards to three weeks' annual holiday.

### Meals.

Day nurses and probationers breakfast at 6.45 a.m.; luncheon is from 9 to 10 a.m.; dinner, 1 to 2 p.m.; tea, 4 to 5; and supper, at 9.15 p.m. All meals are served in the dining-room, no allowances being made to the nurses, supplies for each meal being placed on the dining-room table.

The night nurses have supper at 8.30 p.m., breakfast when they leave the wards at 7.10 a.m., and luncheon before they go to bed at 11.30 a.m.

### Diet for Dyspeptics.

It is impossible to make hard and fast rules for all classes of patients suffering from dyspepsia or indigestion, as while one patient can eat certain food without discomfort, to another it acts like poison. Careful dieting is one of the chief cures, and it is only by watching and observing what kind of food the patient can digest best that those in charge will arrive at the correct treatment for that particular individual.

When dyspepsia is caused by want of nervous tone the meals should be small and frequent, but a patient should not be forced to eat if he has no appetite, but something should be given him to produce the pepsine required to perform digestion. Half an hour before a meal give a small cupful of beef tea; this will give a stimulus to secretion, and the gastric juice is produced, and poured out in time to digest the subsequent meal. Mrs. Hart tells us "this rational treatment of indigestion was discovered by the physiologist Schiff, and it is too little known and practised."

Meals for dyspeptics cannot be too simple; they should be composed of very few dishes, and to eat of one thing only at a time is best for many of them. For instance, a dinner of fish, meat, or game is better than eating smaller quantities of all three. The habit of drinking between and not at meals should be cultivated; this simple rule has been known to cure patients alone without further treatment.

Pastry, rich and highly-spiced foods, must be avoided. The meals should be carefully cooked and daintily served, as the appetite of any patient is often as much tempted by appearance as the actual taste of a dish.

Aim at variety, not in one meal only, but between one day and another. Should the patient enjoy a certain dish, do not overdo him with it by having it too constantly. Never let him know, if possible, what is coming for a meal, as a surprise is good, and dwelling on the future meal may cause an imaginary dislike to it.

Tea to some patients acts as poison, but should any be able to take it be careful to make it with fresh water that boils fast, and allow the leaves to infuse but three minutes. The little teapots with strainers that lift out simplify these matters considerably. People who use their brains much often suffer from indigestion; this can be much reduced by their eating sparingly of heavy foods, making their meals of fish, eggs, vegetables, fruit, and milk; and last, but not least, good wholemeal bread, toast, or crust of white bread.

Peptonised and malted foods are often prescribed for acute cases of dyspepsia, and these are much better prepared at home, where good materials can be procured, than by having resort to patent preparations. Peptonised milk is easily done by heating half a pint with half a gill of water to a temperature of 140 deg.; then mix in one teaspoonful of liquor pancreaticus and 10 grains of bicarbonate of sodium. Put this for one hour and a half in a covered jar, and stand it in a warm place; then boil it for a few seconds and serve.

If cold milk peptonised is required, it need only be mixed, say one pint of milk with half a pint of water, 20 grains of bicarbonate of sodium, and three teaspoonfuls of liquor pancreaticus. Put this for four hours in a room where the temperature is 65 deg.; it will then be ready for use.

Black coffee should be avoided by dyspeptics; it hinders digestion if taken after dinner, but ordinary coffee made in a percolator, or boiled for one minute, many patients can take and find it a very stimulating beverage. Cocoa is nourishing, and that made from nibs is more stimulating than many of the patent powders, and patients can digest it where they could not those that contain a large quantity of starch.

The following are a few choice dishes for patients suffering from dyspepsia or ordinary indigestion:—

Cream of Chicken.—Take the breast and leg of a raw chicken, scrape off the meat, chop it finely, then pound it and rub through a fine wire sieve. To two ounces of this add two raw yolks of eggs, a good pinch of salt, and dust of pepper, mix well together in a large basin, add a quarter of a pint of whipped cream, and the white of three small eggs that have been whipped stiffly with a pinch of salt. Butter some small soufflé or china cases, and fill them three parts full with the mixture; then bake them in a moderate oven for 12 or 15 minutes.

Fillets of Plaice.—By some physicians plaice are considered more digestible than sole. They must be perfectly fresh, and kept in cold water for two hours before using. Remove the fillets by passing a sharp knife down the centre of the back and working the fillets off by keeping the knife close to the bone. If large, divide the fillets in two, dry them, and place them on a well-buttered tin; strain the juice from a large lemon over them; cover with a well-buttered paper, and cook in a moderate oven for 12 minutes. Then dish them up as cutlets are done, one over the other, and pour the liquor from the tin over them, and sprinkle with finely-chopped parsley.

Fried French Beans.—These must be boiled first. Cut them as finely as possible, and well wash them; then put them into plenty of boiling water, in which is a tiny piece of soda and some salt; bring quickly to the boil and cook for twenty minutes. If done strain them as dry as possible, and put them in a frying-pan; let them heat and get dry, then add a piece of butter, and move them about with a wooden spoon. Mix in a few drops of lemon juice and a pinch of pepper, turn out into a hot dish, and serve at once.

Strawberry Soufflé.—Rub six ounces of fresh strawberries through a sieve and put them in a clean stewpan with one and a quarter ounces of flour, one ounce of butter, half a gill of cream, and six ounces of castor sugar, a few drops of vanilla essence, also of carmine, to make it a pretty pink colour. Stir together on the fire till it boils, then put in six ounces of fresh strawberries cut into slices, and three whites of eggs that have been whipped stiffly, with a pinch of salt. Pour the mixture into a soufflé dish that is surrounded with a band of buttered paper, and bake in a moderate oven for about half an hour. When cooked remove the paper and put a folded d'oyly round and serve quickly.

### Appointments.

The Princess Mary Convalescent Home, Bognor.—Miss Sibyl Biddulph Pinehard has been appointed Matron of the above home, which is a branch of the East London Hospital for Children, Shadwell, E., at which hospital she was trained. She also studied at the Gordon House Home Hospital and at Charing Cross Hospital. Her appointments have been as follows: Sister of the children's wards at the Royal South Hants Infirmary, Southampton; night sister from 1896 at Shadwell, where for the last two years she has had charge of the Beckford ward for infants. Miss Pinehard is a member of the Royal British Nurses' Association.

The Eversfield Hospital, St. Leonards-on-Sea.—Miss E. Norton Coleman has been appointed Matron of the above hospital. She was trained, and afterwards staff nurse, at the London Hospital, E., and she was subsequently night superintendent for two years at the Chelsea Infirmary.

### "THE HOSPITAL" CONVALESCENT FUND.

We have much pleasure in acknowledging 2s. 3d. from G. G. for this fund.

### The Book World for Women and Nurses.

[We invite Correspondence, Criticism, Enquiries, and Notes on Books likely to interest Women and Nurses. Address, Editor, THE HOSPITAL (Nurses' Book World), 28 & 29, Southampton Street, Strand, London, W.C.]

THE CARE OF THE SICK. By Dr. TH. BILLROTH, Professor of Surgery in Vienna. Translated by J. BENTALL ENDEAN, with portrait and 52 illustrations. Pp. 333. Fifth edition. (Sampson Low, Marston, and Co. Price 2s. 6d.)

This edition of Dr. Billroth's excellent manual, revised and enlarged, is a very satisfactory book, and will stand high among the many excellent handbooks on nursing which have from time to time appeared. The general outlines are very much the same in all books devoted to one subject, but the details differ widely, and it is in these that we recognise the individuality of the writer. Some practical observations on bedsores in the commencing chapters of the book are followed by several excellent hints for their avoidance. One method consists in procuring a water pillow almost the width of the bed, and filling it to such a degree that when the buttocks are disposed on its lower end the upper part swells and fills in the hollow of the loins, thus at once taking all weight off the affected parts. Of course the space between the water cushion and the ends of the bed are suitably filled in with cushions. Some valuable pages are devoted to baths and their administration, followed by a chapter on effusions, damp frictions, packings, and local wet dressings. Chapter IV. deals with surgical nursing, the preparation of instruments, dressings, sponges, &c., and disinfecting remedies. Here the reader will find much food for reflection, as the subjects treated of are among the most important in the book. Typhoid and other fevers occupy a prominent position, and we are glad to note what emphasis is laid on such simple but too often neglected duties as a softened light, quietness, the rinsing and cleansing at frequent intervals of the mouth, and daily washing all over with tepid water. The position, too, in fevers is described, and the importance of repeated change. Towards the close of the volume are found "general directions" for nursing the mentally diseased. "In this state," says Dr. Billroth, "no preaching, no scolding, nor reasoning is of any use—insanity is not to be dispelled by argument." The nurse is advised to humour the patient as far as possible, ascertain his favourite occupations to promote and superintend them, and stimulate him in every way to an interest in his surroundings. Occupation and work are the most important means for strengthening the body and diverting the mind of the insane. The book ends with a chapter on injuries, poisoned wounds, hæmorrhages, resuscitations, and poisoning—a useful category, and one which the reader will do well to take to heart, for preparation to meet any of the emergencies associated with such casualties greatly enhances a nurse's value.

COPENHAGEN AND ITS ENVIRONS. Published by the Danish Tourist Society. Price 1s. 6d. (London: Simpkin, Marshall, Hamilton, Kent, and Co. 1898.)

The above guide book has been sent us for review, and we recommend it to any of our readers who may be proposing to pass their holidays in the Scandinavian Isles. To such persons the little book has much to commend it, being arranged in an extremely handy form, and in such a manner as to make it a comparatively easy matter to grasp quickly the principal objects of interest in the interesting city with which it deals. "The Sights of Copenhagen" are alphabetically arranged in a concise and ingenious tabular form, which redounds commendably to the editor's credit.

### Presentations.

MISS CROUCHLEY, matron of the Metropolitan Asylum, Leavesden, was, on the 1st inst., presented with a very handsome single-stone diamond ring on the occasion of her leaving Leavesden for Claybury. The Chaplain (the Rev. J. R. B. Watson), in a speech both humorous and touching, made the presentation in the name of the entire staff, as a token of the great affection and respect in which Miss Crouchley was held by all, and as an expression of sincere sorrow and regret at her departure after four and a half years' work at Leavesden. Dr. Elkins, medical superintendent, added a few words testifying to the very able and efficient way in which Miss Crouchley had fulfilled her duties. A few days later the committee of the asylum presented Miss Crouchley with a very handsome set of silver backed brushes, comb, and hand-glass as a mark of their esteem and appreciation.

ON September 19th, Miss H. Hudson, the matron of the Bury Sanatorium, was the recipient of several handsome gifts on the occasion of her leaving Bury before her marriage with Mr. J. B. Godfray, of Detroit, America. The hospital committee and medical officer (Dr. Howorth) gave a silver tea service; the nurses, a Crown Derby sardine dish and sugar stand; Nurse Clara Berry, a bridescake; the domestics, a gold brooch. The superintendent and nurses of the Private Nursing Institution, Stockport, presented her with table linen. The various officers of the Stockport Isolation Hospital, where Miss Hudson has frequently undertaken temporary duty, gave a number of useful gifts.

NURSE CLARKE, who for the past five years has been employed as district nurse under the Stourport and Wilden Nursing Society, was presented with the sum of £12 on the occasion of her leaving in order to take up work in London. The gift was accompanied with a letter from a lady taking an important position in the association, stating how much Nurse Clarke and her work had been appreciated by her patients and employers, and with what pleasure they had subscribed together to give her some token of it.

AFTER seventeen years' service as matron of the Reigate and Redhill Cottage Hospital, Miss Stanton has resigned owing to failing health. After the annual general meeting Miss Stanton's presence was requested in the board-room, and the Chairman, expressing the regret felt by the committee at her resignation, and also the estimation in which her work at the hospital was held, gave her a cheque for £105 and an illuminated address.

ON the 6th inst. Miss Evans, Superintendent of the Gloucester District Nursing Society, was presented by Dr. Spence (the Dean of Gloucester) with a gold watch and chain and an arm-chair. The presentation was made on behalf of the subscribers upon her resignation. Her nurses and colleagues gave Miss Evans a Wedgewood biscuit-box.

AT the quarterly meeting of the Committee of the Jubilee Nursing Association, Buckle, N.B., the Rev. Alexander Miller presented Nurse Tait, who was leaving the service of the association, with a dressing case. Nurse Wood, of Campsie, succeeds Nurse Tait.

### Minor Appointments.

INFECTIOUS DISEASES HOSPITAL, NORTHFIELD, BIRMINGHAM.—Miss Ada Lorton, who was trained at the City Hospital, Birmingham, was appointed Sister-in-Charge of this hospital on the 8th inst. She was at one time assistant nurse at the Solihull Union Infirmary, and, later, charge nurse at the Infectious Hospital, Witton. Miss Lorton has recently been engaged in private nursing.

BELFAST ROYAL HOSPITAL.—Miss Jessie Warr was elected on September 30th Night Superintendent of this hospital. She was trained at the Evelina Hospital and at King's College Hospital, London. Miss Warr has also been sister at the Monsall Hospital, Manchester.

FEVER ISOLATION HOSPITAL, UPNEY.—Miss Emily E. Sowell, who was trained at the Pembrokeshire and Haverfordwest Infirmary, was appointed Head Nurse of this institution on the 11th inst.

### For Reading to the Sick.

THE MEANS OF GROWTH.

"Speak unto the children of Israel, that they go forward!"

**Verses.**

Let me then be always growing,
Never, never standing still,
Listening, learning, better knowing
Thee, and Thy most blessed will;
That the Master's eye may trace,
Day by day, my growth in grace.

Then be it so!
For in better things we yet may grow,
Onward and upward still our way,
With the joy of progress from day to day;
Nearer and nearer every year
To the visions and hopes most true and dear!
Children still of a Father's love,
Children still of a home above!
Thus we look back
Without a sigh, o'er the lengthening track.

—*F. R. Havergal.*

You need the lower life to stand upon,
In order to reach up unto that higher;
And none can stand a-tip-toe in that place
He cannot stand in with two stable feet.

—*E. B. Browning.*

**Reading.**

"Grow in grace, and in the knowledge of our Lord and Saviour Jesus Christ."—2 *Pet.* iii. 18.

The very thing we are longing to do, and perhaps mourning over not doing, and perhaps praying every day that we may do, and seeming to get no answer. But when God has annexed a means to the fulfilment of a command, we cannot expect Him to enable us to fulfil that command if we are not using His means. In this case the means are wrapped in another command: "Desire the sincere milk of the word, that ye may grow thereby." Real desire must prove itself by action; it is no use desiring the milk and not drinking it. "Wherefore criest thou unto Me? Speak unto the children of Israel, that they go forward." Let us to-day, and every day henceforth, "go forward," and use in faith and honest earnestness this His own great means of growth. . . . By the word we shall also grow in the knowledge of Christ. The mere surface of this is obvious. For how do we come to know more of anyone whom, having not seen, we love? Is it not by reading and hearing what he has said and written and done? He hath said, "Search the Scriptures; for they are they which testify of Me." . . . "The words which I speak unto you, they are the spirit, and they are life-quickening and continually life-giving words." Jesus Himself has given us this quick and powerful word of God, and our responsibility is tremendous. He has said, "Search!" Now, are we substituting a word of our own, and merely reading them? He did not say, "Read them," but "Search!" and it is a most serious thought for many a comfortable daily reader of the Bible that, if they are only reading and not searching, they are distinctly living in disobedience to one of His plainest commands.—*F. R. Havergal.*

### Death in Our Ranks.

Sheffield has lost a highly-esteemed resident in the death of Miss Corvan, the head of St. George's Home for Nurses, Sandon Place, Broomhall Park. Miss Corvan was the eldest of five sisters, who all became nurses, the daughters of Archdeacon Corvan, of Wexford. She was trained at Winchester and, after several short appointments, finally settled at Sheffield, being lady superintendent of the Nurses' Home and Training Institution, in Glossop Road, for eleven years. At that date she opened the St. George's Home, in Clarkehouse Road, whence she removed to her present home about two and a half years ago. About thirty or forty nurses are on the private staff, and a district staff of five nurses are employed nursing the sick poor free. Miss Corvan was 58 years of age, and died on the 6th inst.

### Notes and Queries.

**The contents of the Editor's Letter-box have now reached such unwieldy proportions that it has become necessary to establish a hard and fast rule regarding Answers to Correspondents. In future, all questions requiring replies will continue to be answered in this column without any fee. If an answer is required by letter, a fee of half-a-crown must be enclosed with the note containing the enquiry. We are always pleased to help our numerous correspondents to the fullest extent, and we can trust them to sympathise in the overwhelming amount of writing which makes the new rules a necessity.**

**Every communication must be accompanied by the writer's name and address, otherwise it will receive no attention.**

*"Hospital" Convalescent Fund.*

(25) Please give me particulars as to The Hospital Convalescent Fund.—*Nurse Thompson.*

See "Mirror" of September 17th, page 211.

*Lying-in Homes*

(26) I should be glad to hear of some small lying-in home where I could be trained in monthly nursing. I do not want to go in a hospital. —*Jim.*

The East-end Mothers' Home, Commercial Road, E.

*Prison Warders.*

(27) Kindly inform me where to apply for a post in a prison as a warder?—*J. H.*

A man wishing to obtain a situation as prison warder should apply either by letter or personally, to the governor of the prison nearest his residence for the necessary papers, which will at once be sent to him. When he has filled up the form of questions with his answers, he must forward that paper to the Prison Commissioners at the Home Office. If they are satisfied with his answers they will instruct the governor of the prison he first applied to to take him through the regular examinations. The governor will then send him the details of these examinations, and will give him 14 days to prepare himself. He will then have to attend personally at the prison and go through the examinations, which include a medical diagnosis of his state of health. The results will be forwarded to the Home Office, and, if satisfactory, he will receive an appointment to some special prison from the Commissioners.

*Physicians' Fees.*

(28) Is there a rule laid down that a physician must charge a fee of £2 2s. a visit to rich and poor alike at his house in town or country?—*A. G. W.*

There is no rule.

*Physical Culture.*

(29) Can you give me any information of the "Ling or Swedish" system of gymnastics? 1. Where is the best place to study—Paris, London, or Stockholm; and how long does an intelligent pupil require to learn it; and how do medical men regard it? 2. If there is a book on the subject, please send it to me.—*C. H. T. (Melbourne).*

The best place to acquire a thorough knowledge of Ling's system is "The Royal Institute of Gymnastics," Stockholm. The course there is of two or three years' duration, and comprises a thorough instruction in anatomy, physiology, kinesiology, and practical and theoretical gymnastics. There is also an excellent school, we believe, in London, founded on the same principles, viz., Madame Bergman-Osterberg's Physical Training College, near Dartford, Kent. The medical profession regards this system as a valuable physical training; but the exercise for curative purposes should always be studied under the direct supervision of a medical man, and in no case ought they to be prolonged. 2. We send you "Physical Culture," by A. E. Turner (Simpkin, Marshall), price 2s., which is fully illustrated and easy to understand; and there is also "School Gymnastics on the Swedish System," by Dr. Broman (John Ball and Sons, Great Titchfield Street, W.)

*Analyst.*

(30) Can you give me the name and address of a reliable analyst, as I am desirous of finding out all the ingredients in a healing lotion for wounds? Is this likely to be expensive?—*F. E. B.*

We advise you to consult Dr. Paul, analyst, of 13, Fenchurch Avenue, E.C. If you write to him in the first case he would tell you what his charge would be.

*Nursing Lectures in Paris.*

(31) Can you assist me to ascertain whether there are in Paris courses of lectures on surgical or obstetrical subjects, also practical dispensary work, open to a graduate nurse who is to spend the winter in Paris and is desirous to continue improving herself?—*S. E. S.*

We advise you to apply to the Hertford British Hospital, Rue de Villiers, Levallois-Perret, Paris, where they might be induced to admit the nurse to their lectures.

*Lady Roberts' Nurses and Lady Dufferin's Fund.*

(32) Could you tell me to whom to apply for particulars about Lady Roberts' Nurses in India, and also about the Dufferin Fund Nurses.—*M. G. A. W.*

Lady Roberts chooses her own nurses. For particulars of the Dufferin Fund apply to Miss Edith Heather Bigg, Assistant Secretary, 14, Radnor Place, Hyde Park, London, W.

*Stewardess' Berth.*

(33) How can I obtain employment as stewardess on a large ship plying to and from New York?—*Stewardess.*

Apply to the secretaries of the shipping companies. For addresses see daily papers.

**ANSWERS.**

*Address Wanted.*

(5) A correspondent kindly informs us that D. H. Evans and Co., Oxford Street, W., and all leading drapers and outfitters, supply the Alpine wool garments required by F. H.

